# Intraperitoneal nivolumab for malignant ascites in patients with advanced gastrointestinal or pancreaticobiliary tract cancer

**DOI:** 10.1007/s10147-025-02930-y

**Published:** 2025-12-10

**Authors:** Hsiu-Tzu Wang, Yung-Luen Yu, Wen-Jyi Lo, Mei-Chen Lin, Chien-Lun Chu, Chia-Yu Chen, Sing-Ting Wang, Chang-Fang Chiu, En-Jia Bai, Li-Yuan Bai

**Affiliations:** 1 Division of Hematology and Oncology, Department of Internal Medicine, China Medical University Hospital, 2, Yude road, North district, Taichung, 404327 Taiwan; 2 Institute of Translational Medicine and New Drug Development, China Medical University, Taichung, 406040 Taiwan; 3 Graduate Institute of Biomedical Sciences, China Medical University, Taichung, 406040 Taiwan; 4 Center for Molecular Medicine, China Medical University Hospital, Taichung, 404327 Taiwan; 5 Department of Medical Laboratory Science and Biotechnology, Asia University, Taichung, 413305 Taiwan; 6 Cancer Center, China Medical University Hospital, Taichung, 404327 Taiwan; 7 College of Medicine, School of Medicine, China Medical University, Taichung, 406404 Taiwan; 8 Bachelor of Science in Kinesiology, Exercise Science, The University of Texas at Austin, Austin, TX 78712 USA

**Keywords:** Malignant ascites, Gastrointestinal, Pancreaticobiliary, Intraperitoneal, Nivolumab, Cytokines

## Abstract

**Background:**

Malignant ascites occur in 10–15% of patients with gastrointestinal tract cancers. The abundance of immune cells in the peritoneum and ascitic fluid, along with the immunosuppressive environment created by cancer cells, suggests the potential utility of intraperitoneal (IP) immune checkpoint inhibitors for controlling malignant ascites.

**Methods:**

Patients with gastrointestinal or pancreaticobiliary tract cancer and cytologically confirmed malignant ascites received IP nivolumab. Twenty mg of nivolumab diluted in 100 mL of saline was infused into the peritoneal cavity over 10 min following paracentesis. IP treatment was repeated after each subsequent paracentesis until deemed ineffective by the treating physician, upon the occurrence of unacceptable toxicity, or discontinued at the patient’s request. This study was registered at ClinicalTrials.gov (NCT05745233).

**Results:**

The median age of the nine enrolled patients was 55 years. Underlying malignancies included pancreatic (n = 4), biliary tract (n = 3), and gastric cancers (n = 2). After a median of 3 (range: 2–5) treatment cycles, seven patients (77.8%) showed a clinical response, as evidenced by reduced ascitic fluid and prolonged intervals between paracenteses. The only adverse effect observed was grade 1 tenderness at the puncture sites. Reduction in tumor cell count in ascites, rather than changes in the total lymphocyte count or lymphocyte subpopulations, correlated with clinical response. Responders consistently exhibited increased vascular endothelial growth factor A and decreased interleukin-1α levels following nivolumab administration.

**Conclusion:**

Intraperitoneal administration of nivolumab effectively controlled malignant ascites with minimal adverse effects. However, further validation in a larger cohort is required.

**Supplementary Information:**

The online version contains supplementary material available at 10.1007/s10147-025-02930-y.

## Introduction

Malignant ascites develop in 10–15% of patients with gastrointestinal cancers at some stage of the disease [1], including 8–18.3% of patients with gastric cancer [2, 3], 15–21% of those with advanced pancreaticobiliary cancers [3, 4], and approximately 4.0% of patients with esophageal cancer. Several factors contribute to the formation of malignant ascites, including obstruction of lymphatic drainage by tumor cells, altered vascular permeability, hormonal mechanisms, and sodium retention mediated by activation of the renin–angiotensin–aldosterone system [5]. Malignant ascites is associated with poor prognosis, with a median survival of 1–4 months [6]. Systemic chemotherapy is generally ineffective against malignant ascites, primarily because of inefficient intravenous drug delivery to peritoneal carcinomatosis deposits and low drug concentrations in the ascitic fluid. This unfavorable therapeutic gradient is largely attributable to high interstitial fluid pressure, caused by the dense extracellular matrix surrounding cancer cells and the disorganized vasculature resulting from tumor angiogenesis [7, 8]. These immature and hyperpermeable blood vessels not only promote ascites accumulation but also increase interstitial pressure, thereby impeding the penetration of anticancer drugs and immune cells into the tumor microenvironment [9, 10].

Management options for malignant ascites include dietary modifications, diuretics, on-demand paracentesis, drainage catheter implantation, peritoneovenous shunting, intraperitoneal (IP) chemotherapy [11, 12], IP administration of picibanil (OK-432) [13–15], cytoreductive surgery combined with hyperthermic IP chemotherapy [16, 17], and systemic anticancer therapies. IP therapy offers several advantages, including high local concentrations of anticancer drugs, direct exposure of free cancer cells and metastatic nodules on the peritoneal surface, and reduced systemic toxicity. However, the response rates are variable and often temporary. Additionally, some IP therapies are associated with significant adverse effects, such as bowel surface toxicity, abdominal pain, peritonitis, and catheter obstruction [13, 18].

The peritoneum is considered a lymphoid organ with distinct immunological characteristics [19], containing abundant dendritic cells (DCs), a predominance of CD8 + over CD4 + T cells, and various soluble immunomodulatory factors [20, 21]. However, in patients with malignant ascites, cancer cells disseminated across the peritoneum can inactivate DCs, induce T cell exhaustion, and overexpress programmed cell death ligand 1 (PD-L1), thereby evading cytotoxic T cell responses [20, 22, 23]. In addition, angiogenic factors promote the accumulation of regulatory T cells and suppress effector T cell functions [24]. Together, tumor-driven angiogenesis and the upregulation of immune checkpoints such as programmed cell death protein 1 (PD-1) and cytotoxic T-lymphocyte-associated protein 4 (CTLA-4) by cancer cells contribute to a highly angiogenic and immunosuppressive microenvironment [10]. These findings support the rationale for IP immunotherapy as a strategy to restore anticancer immune activity and overcome immune evasion. However, prior clinical studies of IP immunotherapy using agents such as tumor necrosis factor (TNF) or interferon have yielded inconsistent ourcomes [25]. Immune checkpoint inhibitors (ICIs) activate immune cells with anticancer properties, including cytotoxic T cells, natural killer cells, and macrophages. Theoretically, IP administration of ICIs represents an appealing strategy for treating malignant ascites, considering the distinct immunological features of the peritoneum and ascitic fluid.

We previously reported a case of a pancreatic cancer patient with refractory malignant ascites who responded favorably to IP nivolumab administration [26]. Building on this observation, we prospectively treated additional patients with malignant ascites and analyzed the ascitic fluid using flow cytometry to assess the immunological impact and clinical efficacy of IP ICIs. Here, we present the findings of nine patients treated with IP nivolumab.

## Patients and methods

### Patients

This prospective study was conducted at China Medical University Hospital between January 1, 2021, and January 31, 2023. Patients with gastrointestinal or pancreaticobiliary tract cancer and massive malignant ascites were eligible for screening. The presence of malignant ascites should be confirmed cytologically or pathologically. The study protocol was approved by the Research Ethics Committee of China Medical University and Hospital, Taichung, Taiwan (CMUH107-REC1-180). This study was registered at ClinicalTrials.gov (NCT05745233).

### Treatments

Patients with symptomatic ascites who fulfilled the inclusion criteria underwent paracentesis as clinically indicated. During paracentesis, 20 mL of ascitic fluid was collected for flow cytometry and cytokine analysis. After paracentesis, patients were administered an IP infusion of nivolumab (20 mg diluted in 100 mL saline) over 10 min. The paracentesis catheter was removed immediately after nivolumab infusion was completed. After paracentesis, patients remained in bed with position changes every 20 min for 2 h: supine, on the right side, supine, on the left side, head high and feet low, and head low and feet high. Ascites was assessed by the treating physicians. Responders were defined as patients with increased paracentesis interval, whereas non-responders were patients were defined as patients with decreased paracentesis interval. IP treatment was repeated after each subsequent paracentesis until the treatment was deemed ineffective by the treating physician, unacceptable toxicity occurred, or stopped at the patient’s request.

### Evaluation of toxicity

Physical examination, blood pressure, heart rate, and respiratory rate were recorded before and after each paracentesis and IP infusion of nivolumab. Any adverse events were documented and graded according to the National Cancer Institute Common Terminology Criteria for Adverse Events, version 5.0.

### Analysis of the cellular components of ascites

The cellular components of 20 mL of ascitic fluid, obtained during paracentesis, were analyzed using flow cytometry. Briefly, cells were concentrated by centrifuging the fluid at 2,000 rpm for 10 min. The resulting cell pellets were washed with 3 mL of phosphate-buffered saline (PBS) containing 0.2% bovine serum albumin (BSA) and centrifuged three times. The final pellet was resuspended in 100 µL of PBS with 0.2% BSA. The cells were stained with the following antibodies: CD45 (2D1, Horizon V500, BD Biosciences), CD44 (L178, fluorescein isothiocyanate [FITC], BD Biosciences), CD24 (ML5, APC-H7, BD Biosciences), CD71 (YDJ1.2.2, APC-A750, Beckman Coulter), CD200 (OX104, APC, Invitrogen), and CD326 (epithelial cell adhesion molecule [EpCAM], EBA-1, phycoerythrin [PE], BD Biosciences). The samples were incubated in the dark at 25 °C for 30 min. Erythrocytes were lysed using 2 mL of BD FACS™ Lysing Solution (BD Biosciences). For intracellular staining, cells were then fixed and permeabilized using the FIX & PERM™ Cell Permeabilization Kit (Invitrogen™), following the manufacturer’s instructions, and stained with anti-human cytokeratin-7/−8 antibody (CAM5.2, Brilliant Violet™ 421 [BV421], BD Biosciences). Finally, stained cells were washed once with PBS, resuspended in 200 µL of PBS containing 0.2% BSA, and analyzed using a BD FACSLyric™ cytometer (BD Biosciences, CA). Cancer cells were identified as CD45 −/EpCAM +/Cytokeratin +/CD44 +/CD24 +/CD71 +/CD200 −. Mesothelial cells were excluded based on CD200 expression.

### Analysis of cytokines of ascites

Ascitic fluid samples from six patients (five clinical responders and one non-responder) were available for cytokine analysis both before and after IP administration of nivolumab. Paired ascites samples were analyzed for 19 cytokines, including: epidermal growth factor (EGF), fibroblast growth factor 2 (FGF-2), granulocyte colony-stimulating factor (G-CSF), interferon α 2 (IFNα2), interferonγ (IFNγ), interleukin 1α (IL-1α), interleukin 1β (IL-1β), interleukin 2 (IL-2), interleukin 6 (IL-6), interleukin 12p70 (IL-12p70), interleukin 22 (IL-22), monocyte chemoattractant protein-1 (MCP-1), monocyte chemoattractant protein-3 (MCP-3), macrophage colony-stimulating factor (M-CSF), macrophage-derived chemokine (MDC), macrophage inflammatory protein-1α (MIP-1α), macrophage inflammatory protein-1β (MIP-1β), vascular endothelial growth factor A (VEGF-A), and soluble CD40 ligand (sCD40L). Cytokine levels were measured using the Milliplex® Human Cytokine/Chemokine/Growth Factor Magnetic Bead Panel A/B (Merck KGaA, Darmstadt, Germany), according to the manufacturer’s protocol. All cytokines were measured in triplicate for each sample.

### Statistical analysis

The outcome measure was the clinical response of ascites, defined as a reduction in ascitic fluid volume and a prolonged interval between paracenteses following IP nivolumab treatment. Duration of response was defined as the time interval from the initiation of IP nivolumab until it was deemed ineffective by the treating physician, upon the occurrence of unacceptable toxicity, or stopped at the patient’s request. All statistical analyses were performed using Statistical Product and Service Solutions version 26 for Windows (IBM Corporation, Armonk, NY, USA), with two-sided p-values < 0.05 considered statistically significant.

## Results

### Patient characteristics

Between January 1, 2021, and January 31, 2023, nine patients with malignant ascites secondary to gastrointestinal or pancreaticobiliary tract cancers received IP nivolumab therapy (Table 1). The underlying malignancies included pancreatic cancer in four patients, biliary tract cancer in three, and gastric cancer in two. All patients had previously received systemic therapy for their underlying disease, except for patient number (b). No patient had received any form of local therapy for ascites or pleural effusion.

**Table 1:** Characteristics of patients and ascites content

Patient	Age (years)	Sex	Underlying disease	Other metastatic site	Prior systemic anticancer therapies	Prior local treatment for ascites	Systemic Tx prior to IP Nivo	Systemic Tx cocurrent IP Nivo	Systemic Tx post to IP Nivo	Cancer cells in ascites before first IP (/cumm)	Neutrophils in ascites before first IP (%)	Lymphocytes in ascites before first IP (%)	Lymphocytes in ascites before first IP (/cumm)
a	55	M	PC	–	2	No	Gem + A liposomal irinotecan + 5-FU	–	–	0.258	59	29	249.4
b	53	F	PC	liver	1	No	DCV	–	–	0.517	9	72	30.2
c	52	F	PC	–	2	No	mFOLFIRINOX Gem + A	liposomal irinotecan + 5-FU × 3	–	1.063	2	96	329.3
d	71	M	BTC	lymph nodes	1	No	Gem + A + cisplatin liposomal irinotecan + 5-FU	–	–	1.586	4	37	45.1
e	53	F	PC	–	1	No	Gem + A + oxaliplatin	liposomal irinotecan + 5-FU × 1	–	1.976	1	25	23.8
f	71	F	BTC	liver	3	No	Gem + cisplatin pembrolizumab + lenvatinib LOXO LY3410738	–	–	3.277	7	18	178.7
g	55	M	GC	–	3	No	capecitabine + oxaliplatin docetaxel + ramucirumab FOLFIRI	–	–	24.381	28	19	51.3
h	48	F	GC	–	1	No	Nivolumab + mFOLFOX7 docetaxel + ramucirumab	–	–	4.016	11	39	62.4
i	69	M	BTC	–	1	No	Gem + TS-1	–	–	7.254	7	85	153

Patient	N/L in ascites before first IP	Cancer cells in ascites before first IP (/cumm)	Neutrophils in ascites before first IP (%)	Lymphocytes in ascites before first IP (%)	Lymphocytes in ascites before first IP (/cumm)	N/L in ascites before first IP	Clinical response	Total cycles of IP	Duration of treatment (days)	Cause of termination	OS (diagnosis to death, days)	OS (first IP to death, days)
a	2.0	0.258	59	29	249.4	2.0	Yes	2	28	Patient’s wish	385	35
b	0.1	0.517	9	72	30.2	0.1	Yes	4	48	Expire due to disease	114	49
c	0.0	1.063	2	96	329.3	0.0	Yes	5	64	Expire due to disease	412	108
d	0.1	1.586	4	37	45.1	0.1	Yes	3	38	Patient’s wish	625	55
e	0.0	1.976	1	25	23.8	0.0	Yes	2	29	Expire due to disease	152	55
f	0.4	3.277	7	18	178.7	0.4	Yes	3	40	Expire due to disease	512	41
g	1.5	24.381	28	19	51.3	1.5	Yes	5	37	Expire due to disease	268	29
h	0.3	4.016	11	39	62.4	0.3	No	2	19	Uncontrolled ascites	664	73
i	0.1	7.254	7	85	153	0.1	No	2	10	Uncontrolled ascites	67	11

*A* Nab-paclitaxel, *BTC* Biliary tract cancer, *DCV* Dendritic cell vaccine, *F* Female, *5-FU* 5-Fluorouracil, *GC* Gastric cancer, *Gem* gemcitabine, *IP* Intraperitoneal, *L* Lymphocyte, *M* Male, *N* Neutrophil, *PC* Pancreatic cancer, *Tx* Treatment

The cellular components of ascitic fluid were analyzed by flow cytometry. At baseline, the median cancer cell count was 1.976 (range: 0.258–24.381) cells/µL. The median neutrophil proportion was 7% (range: 1–59%), while the median lymphocyte proportion was 37% (range: 18–96%) with an absolute lymphocyte count of 62.4 (range: 23.8–329.3) cells/µL. The median neutrophil-to-lymphocyte ratio in the ascitic fluid was 0.1 (range: 0–2.0) (Table 1).

### Treatment

All patients received a second dose of IP nivolumab based on symptoms of abdominal fullness, which ranged from 3 to 21 days. The median number of treatment cycles was three (range: 2–5) (Table 1).

All patients tolerated IP nivolumab well. No cases of fever, abdominal cramping, nausea, vomiting, or allergic reactions were observed. The only adverse event was grade 1 tenderness at the puncture sites.

### Therapeutic efficacy

Of the nine patients, seven achieved a clinical response, as evidenced by a reduction in ascites volume and a prolonged interval between paracentesis following IP nivolumab treatment [patients (a) to (g)]. In contrast, ascites in two patients did not respond, and IP therapy was discontinued after two treatment cycles in patients (h) and (I) (Table 1).

**Fig. 1:**
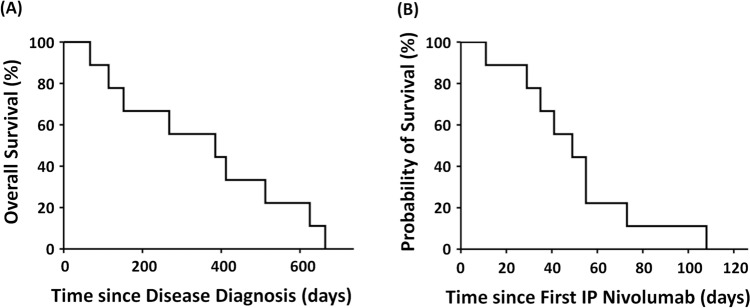
Kaplan–Meier curves for overall survival. **A** From the date of disease diagnosis. **B** From the date of the first intraperitoneal nivolumab

The median number of treatment cycles and duration of IP treatment were 3 and 37 days (range: 10–64 days), respectively. Among clinical responders, IP nivolumab was discontinued due to disease progression and death in five patients and upon patient request in two. The median overall survival from disease diagnosis and from the start of IP nivolumab was 385 days and 49 days, respectively (Fig. 1).

### Tumor cells in ascites decreased in clinical responders

**Fig. 2:**
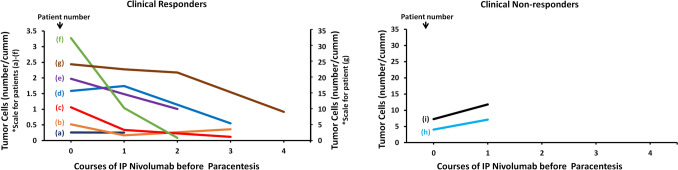
Time course of tumor cell counts in ascites before and after intraperitoneal administration of nivolumab. The left and right panels represent data from seven clinical responders and two clinical non-responders, respectively. Each line represents an individual patient. Tumor cells were analyzed using ascitic fluid collected prior to each administration of intraperitoneal nivolumab

**Fig. 3:**
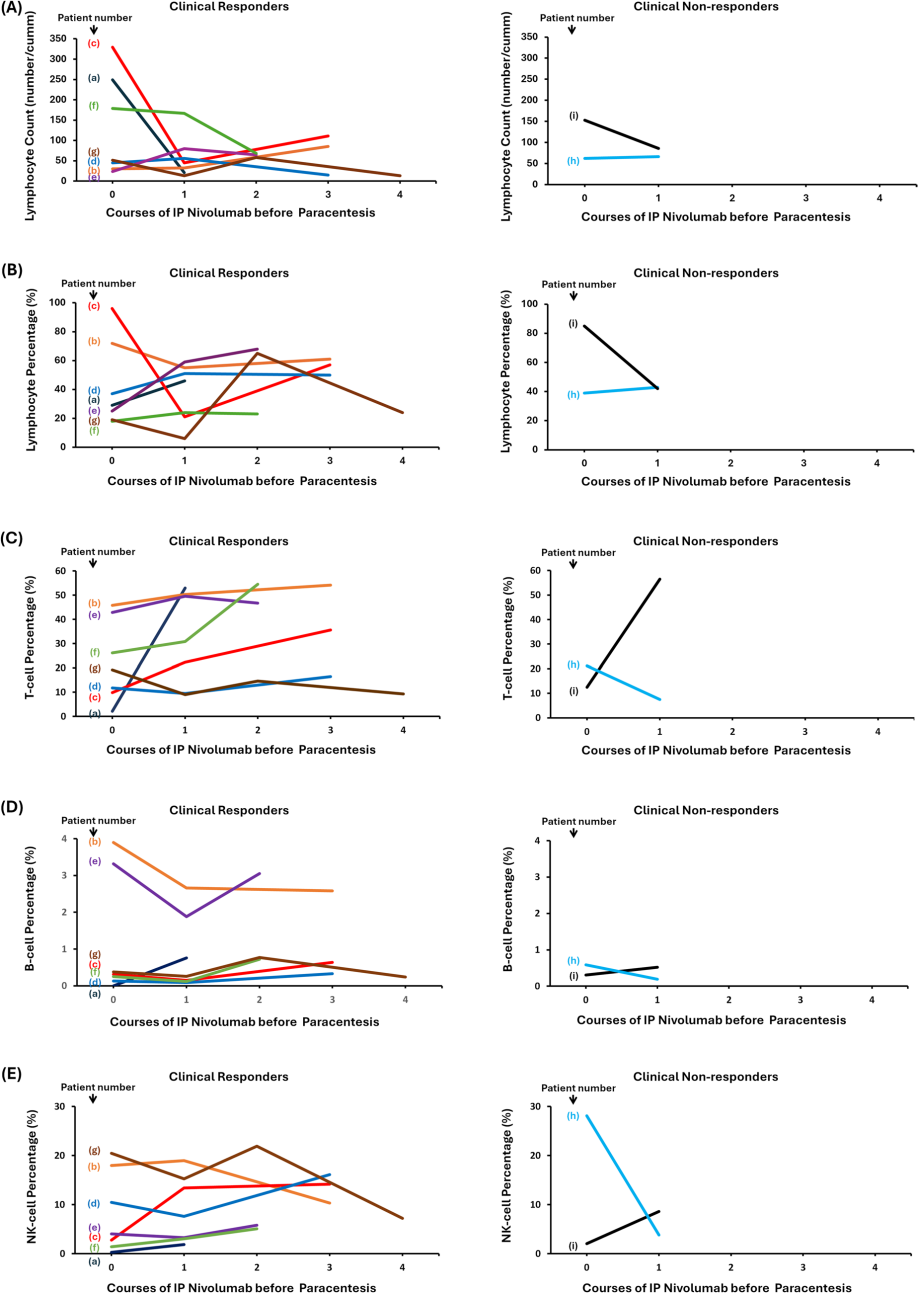
Changes in lymphocyte count (**A**), lymphocyte percentage (**B**), T cell percentage (**C**), B cell percentage (**D**), and natural killer (NK) cell percentage (**E**) in ascites before and after intraperitoneal nivolumab administration. The left and right panels represent data from seven clinical responders and two clinical non-responders, respectively. Each line represents an individual patient. Immune cells were analyzed using ascitic fluid collected prior to each course of intraperitoneal nivolumab

Tumor cell count, neutrophil count, lymphocyte count, and the neutrophil-to-lymphocyte ratio in ascites were analyzed in relation to clinical response. Tumor cell counts decreased in six of the seven clinical responders, except in patient (a). Conversely, increased tumor cell counts were observed in both non-responders (Fig. 2). Changes in total lymphocyte count (Fig. 3A), lymphocyte percentage (Fig. 3A), T cell percentage (Fig. 3C), B cell percentage (Fig. 3D), and natural killer cell percentage (Fig. 3E) in ascites did not correlate with the clinical response.

### Cytokine changes in ascites in clinical responders vs. non-responders

**Fig. 4:**
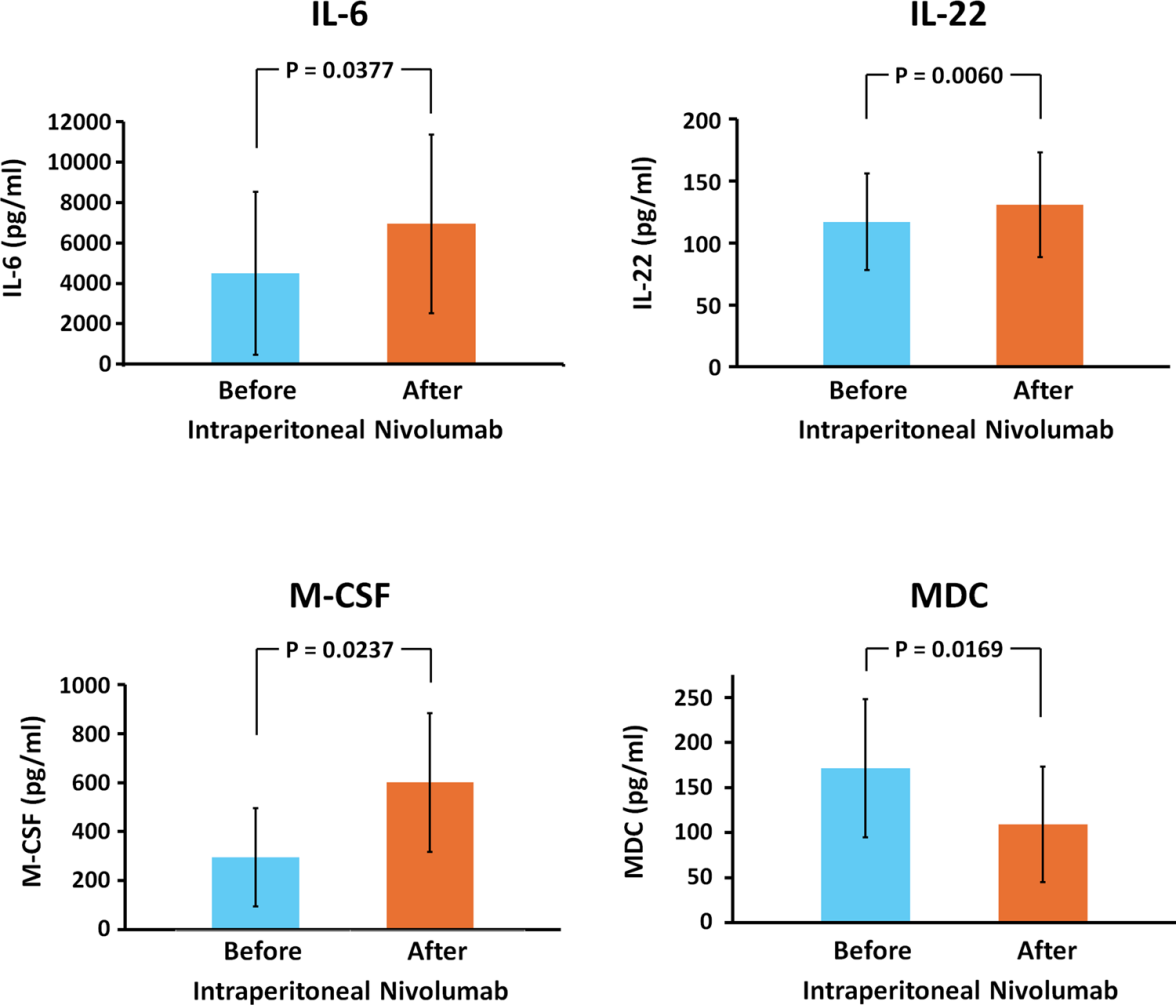
Levels of interleukin 6 (IL-6), interleukin 22 (IL-22), macrophage colony-stimulating factor (M-CSF), and macrophage-derived chemokine (MDC) in ascites before and after intraperitoneal nivolumab administration. Data represent the mean ± standard deviation of three independent experiments

The concentrations of 19 cytokines in ascitic fluid before and after IP nivolumab administration were compared (Supplementary Fig. 1). Among these, IL-6, IL-22, and M-CSF showed significant increases following treatment, while MDC levels decreased (Fig. 4). However, notable interpatient variability was observed.

**Fig. 5:**
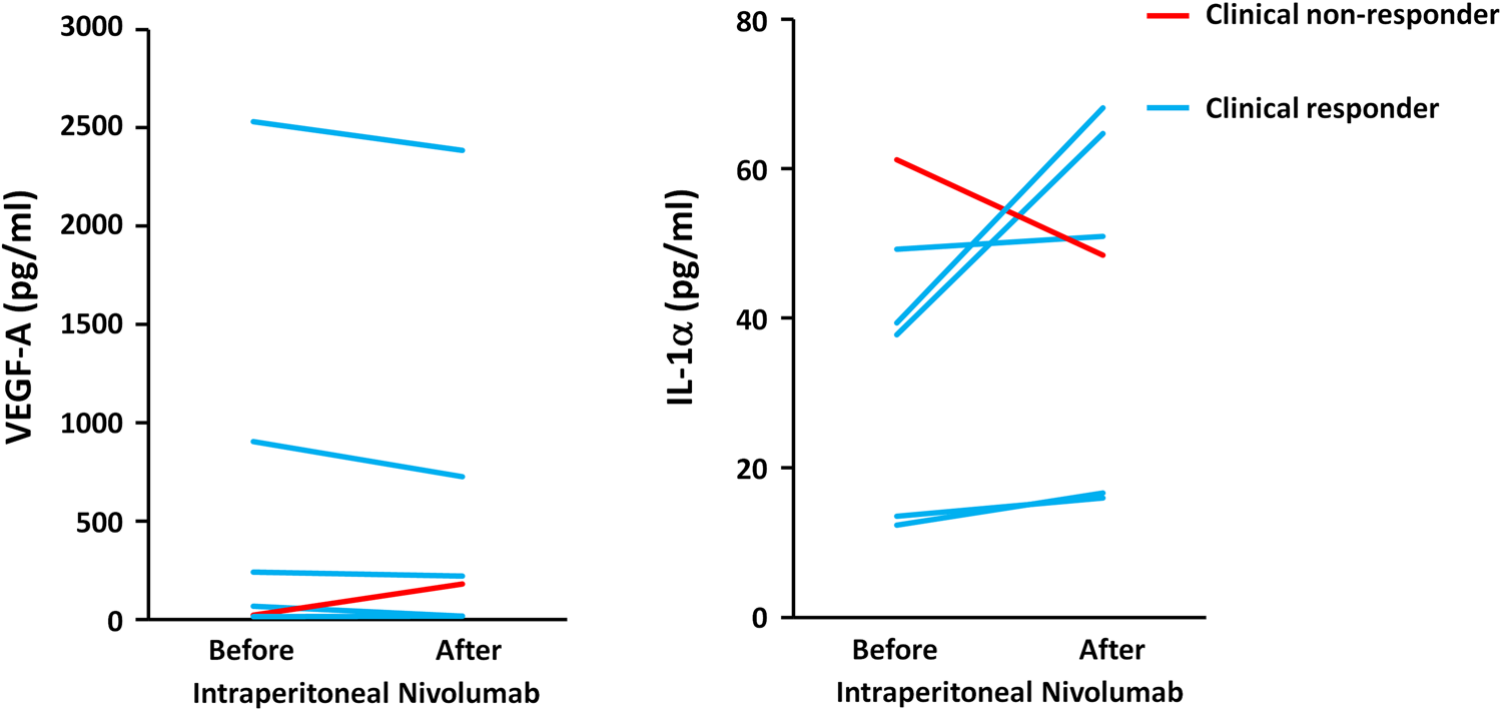
Levels of vascular endothelial growth factor A (VEGF-A) and interleukin 1α (IL-1α) in ascites before and after intraperitoneal nivolumab administration. Each line represents an individual patient

Cytokine profiles were compared between responders and non-responders to assess the relationship between cytokine changes and the clinical response. VEGF-A and IL-1α exhibited consistent trends that distinguished clinical responders from the non-responder (Fig. 5). All five responders showed a decrease in VEGF-A levels, whereas non-responders exhibited an increase following IP nivolumab treatment. Conversely, IL-1α levels increased in the ascites of all clinical responders, in contrast to a decrease observed in the non-responder.

## Discussion

This prospective analysis of nine patients with gastrointestinal or pancreaticobiliary tract cancer demonstrated for the first time that IP nivolumab is effective in controlling malignant ascites, with a clinical response rate of 77%. Notably, the treatment was well tolerated, with no reports of abdominal pain, fever, gastrointestinal upset, or allergic reactions, except mild local tenderness at the site of paracentesis catheter insertion. A reduction in the number of cancer cells in ascitic fluid correlated well with clinical response, underscoring the potential of IP immune checkpoint inhibitors.

Given the unique immune characteristics of the peritoneal cavity and the abundance of anticancer immune cells in ascites, IP immunotherapy has been actively explored since the 1980 s, with varying degrees of success. Agents previously investigated include the immune-stimulator picibanil [13–15], interferon [21, 27], TNF [25], and monoclonal antibodies such as mouse IgG1 targeting the EpCAM [28]. IP administration of picibanil, a penicillin-inactivated, heat-treated preparation of *Streptococcus pyogenes* A3 designed to stimulate NK and T cells, resulted in ascites reduction in 50–70% of patients [13–15]. However, its use is often limited by intolerable adverse effects, with over 90% of patients experiencing abdominal cramping, fever, chills, bowel distention, nausea, and vomiting.IP administration of TNF or interferon has been proposed to enhance the activity of cytotoxic immune cells within the peritoneal cavity. However, their clinical application remains controversial due to notable adverse effects [21, 25, 27].

ICIs have demonstrated anticancer activity against a broad range of malignancies. IP infusion of ICIs directly targets the peritoneal cavity and has the potential to enhance local immune responses. Both pembrolizumab and nivolumab have been administered intraperitoneally in murine tumor models without evident toxicity [29]. In particular, nivolumab was reported to be substantially more effective than dacarbazine in controlling peritoneal metastases of malignant melanoma in mouse models [30]. To date, the IP administration of ICIs for treating malignant ascites has not been established [26, 31, 32]. A phase I trial investigated the combination of IP ipilimumab and nivolumab in 23 patients with peritoneal carcinomatosis secondary to gynecological malignancies [31]. The overall response rate was 18.8% in 16 evaluable patients. Grade ≧3 immune-related adverse events occurred in 2 (8.7%) patients, and the recommended phase 2 dose was nivolumab at 3 mg/kg every 2 weeks plus ipilimumab at 1 mg/kg every 6 weeks. Another phase I/II trial assessed IP nivolumab after debulking surgery and hyperthermic IP chemotherapy in patients with advanced ovarian carcinoma [32]. Of the 17 patients who received treatment, no dose-limiting toxicity was observed in 9 evaluable patients. Therefore, IP nivolumab at 3 mg/kg should be investigated in future studies. The target population in our cohort was different from that of the two studies. In addition, we performed an analysis of ascitic fluid. Although preliminary, these two publications, along with our report, suggest the feasibility and promising role of IP ICIs in patients with cancer.

The VEGF-A levels in ascites were consistently lower in clinical responders than in the non-responder (Fig. 5). VEGF, released by tumor cells, is a well-recognized factor promoting ascites formation [33]. Consequently, several studies have explored the therapeutic potential of anti-VEGF antibodies in managing malignant ascites [33, 34]. Conversely, IL-1α levels in ascites consistently increased in clinical responders, in contrast to the non-responders. IL-1α, produced by epithelial cells, endothelial cells, activated macrophages, and neutrophils, plays a central role in inflammation. Its in vivo functions include promoting collagen synthesis, stimulating fibroblast proliferation, increasing neutrophil recruitment, activating lymphocytes, stimulating the immune system, and inducing fever [35]. Therefore, the observed increase in IL-1α among responders suggests an immune-activating environment induced by intraperitoneal nivolumab.

This study has several limitations. First, this was a small prospective analysis involving only nine patients. The limited cohort not only restricts the statistical power but also undermines the reliability of subgroup analyses, such as cytokine profiling and tumor cell quantification. In the future, the potential of IP ICIs should be validated in larger cohorts using a control arm. Second, there was no standardized interval for paracentesis and IP ICIs; instead, the treatment timing was based on patients’ symptoms. In our study, patients had advanced gastrointestinal or pancreaticobiliary tract cancer, developed symptomatic malignant ascites, and were refractory to or refused standard of systemic anticancer treatment. Our goal was to improve symptoms caused by malignant ascites in hospice care for which paracentesis/IP nivolumab was administered in a symptom-driven manner. However, we acknowledge that a study adopting a protocol-defined administration pattern would be more robust and is essential to determine the value of IP ICIs. Third, we did not analyze predictive biomarkers at baseline to identify patients most likely to benefit from this treatment owing to the limited number of patients. Fourth, the pharmacokinetic of nivolumab in systemic circulation were not assessed. Given the potential for systemic immune activation by absorbed nivolumab from ascites, the future studies should include pharmacokinetic analysis of nivolumab in the systemic circulation.

## Conclusion

IP administration of ICIs appears to be a safe, effective, and quality-of-life-preserving approach for managing malignant ascites in patients with gastrointestinal or pancreaticobiliary tract cancers. Further studies involving larger patient cohorts, malignancies of diverse origins, and biomarker investigations are required to validate and expand upon these findings.

## Supplementary Information

Below is the link to the electronic supplementary material.

Supplementary file1 (TIF 789 KB)

## Data Availability

The data that support the findings of this study are available from the corresponding author upon reasonable request.
