# Supplementary figures and images for: Intraperitoneal nivolumab for malignant ascites in patients with advanced gastrointestinal or pancreaticobiliary tract cancer
Source: Int J Clin Oncol. 2025 Dec 10;31(2):281–91. doi: 10.1007/s10147-025-02930-y (PMC12847151; doi:10.1007/s10147-025-02930-y)

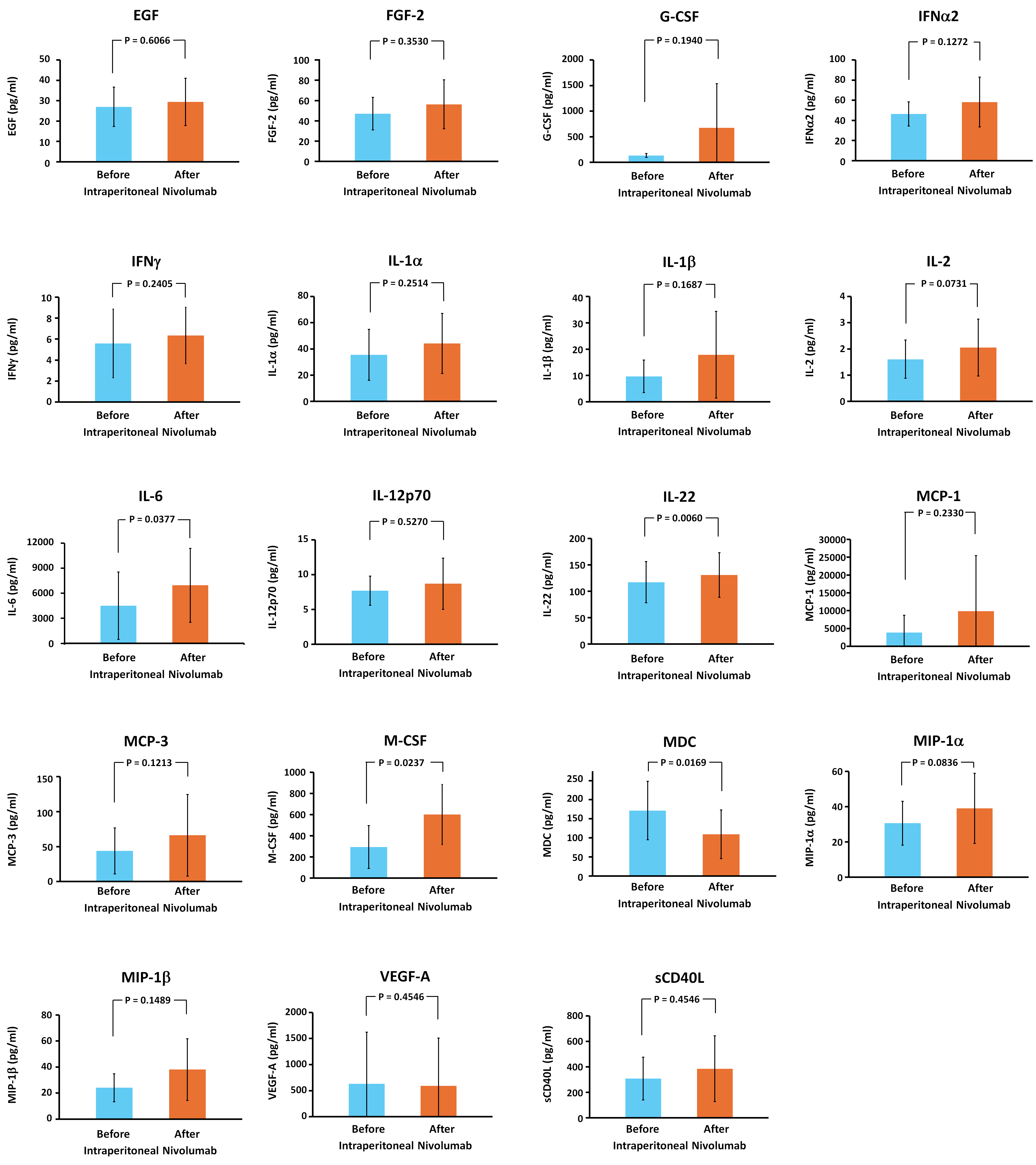

Supplement: Supplementary file 1 — Supplementary file1 (TIF 789 KB) [file 10147_2025_2930_MOESM1_ESM.tif]
